# Advancing Oral Health Equity in the Asia-Pacific Region – A Call to Action

**DOI:** 10.1016/j.identj.2025.103892

**Published:** 2025-09-22

**Authors:** Chun Hung Chu

The Asia Pacific Dental Federation (APDF/APRO), a cornerstone of dental advocacy since 1955, continues to champion oral health equity across one of the world’s most diverse regions. At the 45th Asia Pacific Dental Congress in Taipei, experts convened to address three urgent challenges: the oral health of aging populations, the burden of early childhood caries, and the unmet needs of individuals requiring special care dentistry. This special issue presents their consensus-driven recommendations, offering a roadmap for policymakers, professionals, and communities to transform oral health outcomes.

Asia-Pacific’s rapidly aging population faces a silent crisis. Two-thirds of older adults suffer from untreated tooth decay^1^, while nearly all experience periodontal disease^2^. These conditions worsen chronic illnesses such as diabetes and heart disease^3^, yet oral health remains sidelined in elder care. The APDF’s review calls for universal healthcare systems to integrate oral services for older adults, ensuring affordability and accessibility. Dental professionals must adopt minimally invasive treatments and collaborate with medical providers to address shared risk factors^4^. Educational institutions are urged to embed geriatric care in curricula, while governments must fund community programs that promote preventive care. Without systemic change, aging populations will face compounded health and economic hardships.

Half of Asia-Pacific’s preschoolers endure early childhood caries, yet fewer than 10% receive treatment^5^. Pain and infection from untreated decay harm nutrition, speech, and school performance, perpetuating cycles of disadvantage. The APDF’s experts advocate for universal health coverage to include preventive dental care for children, starting with mandatory annual checkups in kindergartens. Schools should teach oral hygiene and partner with oral healthcare professionals to deliver fluoride therapy^6^. Parents need support to reduce sugary diets and adopt early brushing habits. Dental professionals must shift from surgical interventions to affordable, minimally invasive options like silver diamine fluoride. By prioritizing prevention, the region can spare millions of children from preventable suffering.

Individuals with disabilities in Asia-Pacific often face insurmountable barriers to dental care, from inaccessible clinics to untrained staff. While some countries like Japan^7^ lead in specialized services, others struggle with fragmented systems. The APDF’s review urges governments to enforce disability-friendly clinic standards and expand mobile dental units for remote areas. Universities must train future dentists in special care techniques, while professional bodies should offer continuing education^8^. Collaboration among dentists, caregivers, and social workers is critical to address complex needs. Aligning with the UN Convention on the Rights of Persons with Disabilities, the APDF underscores oral health as a nonnegotiable human right.

These three reviews in this special issue share a common thread: oral health disparities reflect broader societal inequities. Older adults, children, and individuals with disabilities are disproportionately affected by underfunded systems, poor awareness, and fragmented care. Success hinges on collaboration. Policymakers must allocate resources, educators must innovate, and professionals must advocate. The APDF’s legacy of regional cooperation provides a foundation, but progress demands urgency.

Oral health is not a luxury. It is integral to dignity, well-being, and economic productivity. As Asia-Pacific navigates demographic and epidemiological shifts, neglecting oral health will strain healthcare systems and deepen inequalities. The recommendations in this issue are both a blueprint and a moral imperative. Let us act decisively—for the child with untreated decay, the elder unable to eat, and the person with disabilities denied care. Together, we can ensure that oral health becomes a reality for all, not a privilege for some.

The Asia Pacific Dental Federation stands ready to lead this charge. The time for action is now.
